# The impact of after-school soccer exercises on inhibitory control function in children aged 8–11: an fNIRS study

**DOI:** 10.1186/s12887-025-06477-9

**Published:** 2026-01-10

**Authors:** Mingchao Xu, Siyi Sun, Xiaoke Zhong, Yingxu Pan, Wenwu Leng, Hanzhe Chen, Changhao Jiang

**Affiliations:** 1 Institute of Physical Education and Training, Capital University of Physical Education and Sports, Beijing, 100191 China; 2 School of Kinesiology and Health, Capital University of Physical Education and Sports, Beijing, 100191 China; 3 School of Physical Education and Sport Science, Fujian Normal University, Fujian, 350108 China; 4 Xinyu No. Four Middle School, Jiangxi, 338099 China; 5 Tianjin No. Two High School, Tianjin, 300100 China; 6 The Center of Neuroscience and Sports, Capital University of Physical Education and Sports, Beijing, 100191 China

**Keywords:** After-school physical exercises, Soccer, Children, Inhibitory control, fNIRS

## Abstract

**Background:**

Inhibitory control, a crucial higher cognitive function, is closely associated with children’s daily routines and academic performance. After-school physical activities serve as a fundamental avenue for children to participate in sports and also play a pivotal role in enhancing their cognitive development, thereby significantly impacting their overall growth. This study aims to investigate the impact of 12 weeks of after-school soccer exercise on inhibitory control in children aged 8–11 years.

**Method:**

Using a 2 × 2 mixed experimental design, 70 children aged 8–11 were randomly assigned to either an experimental or control group. The experimental group received 12 weeks of extracurricular soccer training, attending 60-minute sessions three times a week, while the control group maintained their regular lifestyle and study routine. To assess inhibitory control performance and prefrontal cortex activation levels, all participants completed the Flanker task, with prefrontal cortex oxygenation levels measured using functional near-infrared spectroscopy (fNIRS) during the task.

**Results:**

Following the intervention, the experimental group demonstrated a significant increase in accuracy on the incongruent task compared to pre-intervention levels. Post-intervention, there was a notable improvement in accuracy on the Flanker task compared to pre-intervention levels, with no significant changes observed in the control group. Additionally, there was a significant increase in blood oxygen response in the right posterior frontal area (R-PFA) and the right dorsolateral prefrontal cortex (R-DLPFC) in the experimental group compared to pre-intervention levels, while no significant changes were observed in the control group. Pearson correlations indicated a significant association between activation in the R-PFA and R-DLPFC and improvements in accuracy resulting from post-intervention soccer training.

**Conclusion:**

Our research hypothesis is corroborated by the study findings, indicating that involvement in extracurricular physical exercise within a school environment may impact the inhibitory control function of children. It is suggested that the right prefrontal cortex may have a pivotal role in enhancing inhibitory control function through physical activity.

## Introduction

The inhibitory control function is essential for completing tasks as it helps suppress self-emotions, desires, impulses, and other interferences [1]. Belonging to the executive functions category, this advanced cognitive function is uniquely human, setting us apart from other species [2]. This function can be categorized into interference inhibition and response inhibition, based on cognitive processes and neural mechanisms [3]. The development of inhibitory control is closely linked to the maturation of the prefrontal cortex [4]. Research suggests that children may face cognitive impairments and delays during rapid cognitive development, impacting their academic performance, mental and physical health, social adaptation, and overall quality of life in adulthood [5, 6]. Studies suggest that interventions can boost the development of children’s inhibitory control function [7].Therefore, research on the development and promotion of children’s inhibitory control function continues to be a current focus.

Methods to enhance inhibitory control function include behavioral activities, neural regulation, and medication interventions [8–10]. Physical activity has been shown to effectively promote the development of inhibitory control in children [11]. Research indicates that the effects of interventions vary based on the type of exercise, timing, location, and target group [12, 13]. In China, after-school physical exercise is increasingly used to encourage children’s participation in sports [14]. Improving inhibitory control through extracurricular physical activities can have positive effects on children’s behavior [15]. Therefore, utilizing after-school physical exercise to enhance children’s inhibitory control development is a pressing issue to be addressed.

Research indicates that participating in cognitive physical activities can bring about significant changes in children’s brains, ultimately enhancing their cognitive functions [16]. Soccer involves not only aerobic exercise but also open skills that demand sensory perception, active control, and motor coordination [17]. Studies have demonstrated that open skills can effectively enhance the development of executive function in children, regardless of the intervention duration [18]. Moreover, congruent soccer practice has been shown to improve children’s focus and cognitive skills [19]. Additionally, evidence shows that brief periods of intense small-scale soccer matches can boost inhibitory control and attention linked to neurophysiology [20].Therefore, selecting soccer as an intervention method assists in developing a better understanding of the effects of after-school physical activity on inhibitory control function.

Inhibitory control function can be assessed using behavioral tasks and brain imaging techniques. Studies have shown a relationship between inhibitory control function and activation of the prefrontal cortex [21]. Neuroimaging research demonstrates heightened activity in the right prefrontal cortex, specifically the dorsolateral prefrontal cortex (DLPFC), during tasks involving response inhibition and interference suppression [22]. Furthermore, the right posterior frontal area (R-FPA) is linked to elevated cognitive control functions and is engaged in activities requiring attention and inhibitory control [23, 24]. Research on children’s inhibitory function development through physical activity found that improvements in executive function were linked to increased prefrontal cortex activity related to physical activity [25]. While a Previous study indicate that single sessions of open-skill exercises enhance children’s inhibitory control more than closed-skill exercises, yet without a significant increase in prefrontal cortex activation levels [26].

Functional near-infrared spectroscopy (fNIRS) technology is commonly used to investigate the brain mechanisms involved in inhibitory control, particularly in children [27]. Unlike functional magnetic resonance imaging (fMRI) and electroencephalogram (EEG), fNIRS offers a natural and non-invasive approach to studying the neural basis of inhibitory control [28]. It is well-suited for examining individual brain development in children and can be used to explore how soccer affects children’s inhibitory control function [29, 30]. Overall, fNIRS has become a popular and valuable tool in neuroscience due to its non-invasiveness, affordability, and portability.

This study aims to examine the impact of 12 weeks of extracurricular soccer exercise on inhibitory control abilities in school-age children, investigating the association between soccer activities and inhibitory control development as well as changes in brain activation regions. The hypothesis posits that participants in the experimental group will demonstrate elevated brain activation in the right prefrontal cortex and exhibit superior performance on behavioral tasks relative to the control group.

## Materials and methods

### Participants

This study employed repeated measures analysis of variance, determining sample size using G*power software. With an effect size of 0.25, α of 0.05, power of 0.95, and 2 measurements, the minimum required sample size was 54. However, to account for potential participant dropout and test feasibility, 70 children aged 8 to 11 were recruited from a primary school in Beijing, with 37 being male and all having normal or corrected vision. Exclusion criteria included neurological or psychiatric disorders, cardiovascular diseases, and current medication use. Data analysis involved 69 participants, as one from the experimental group withdrew for not meeting study requirements.

### Study design

This study employed a mixed research design, incorporating two dimensions: time (pre-test and post-test) and group (control group and experimental group). Group was used as a between-subject variable, while time was treated as a within-subject variable. The study was conducted as a randomized controlled trial, with participants randomly assigned to either the experimental or control group using a card selection method.Gender distribution between groups was balanced, with no significant difference (χ^2^ = 0.381, *p* = 0.537).The study’s primary outcome measures include accuracy and reaction time in incongruent trials of the Flanker task, reaction time and accuracy of the Flanker effect, and prefrontal cortex oxygenation changes during task execution, assessed using functional near-infrared spectroscopy (fNIRS). Secondary outcome measures encompass performance in reaction time and accuracy of congruent trials across various trial types.

### Experimental procedure

**Fig. 1:**
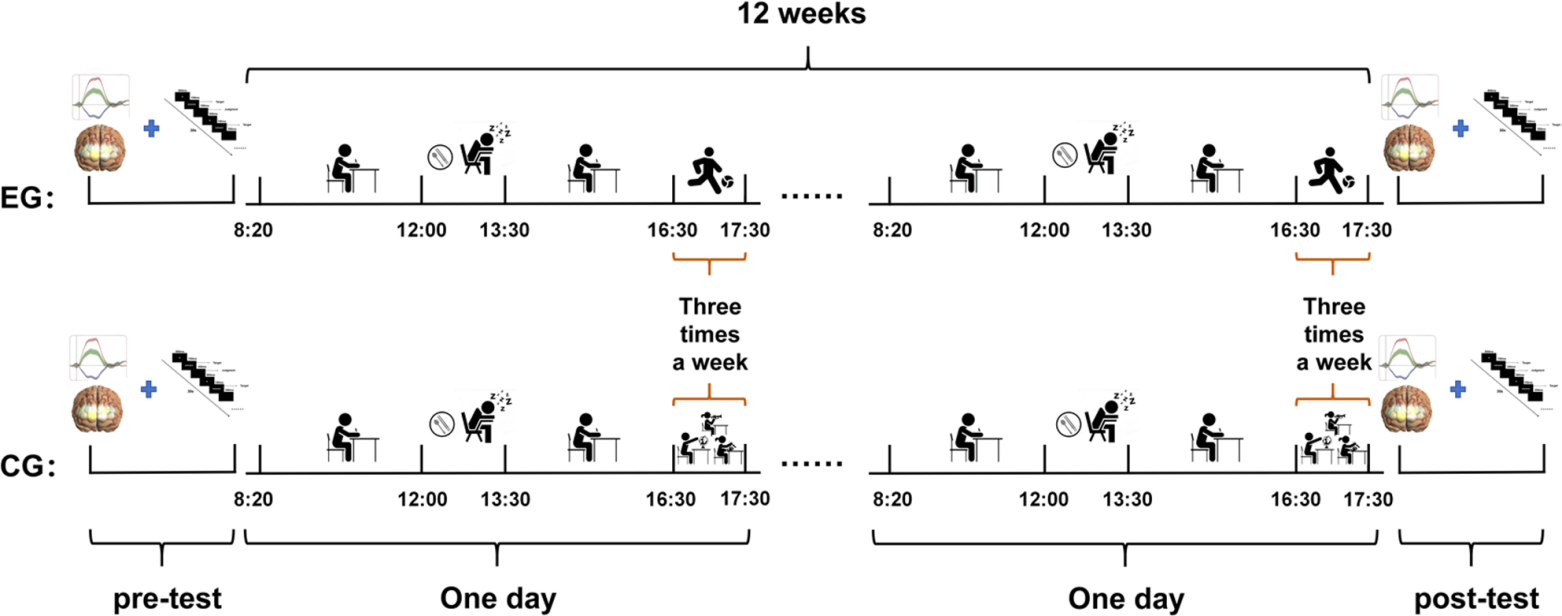
Schematic representation of the two experimental conditions. EG: Experimental Group. CG: Control Group

The experimental group engaged in a 12-week campus football training program, consisting of three weekly sessions. The intervention took place during after-school activities, each lasting 60 min, including a 10-minute warm-up, a 10-minute cool-down, and 40 min of football-specific training. This training was overseen by a professional football coach holding AFC C-level certification, focusing on skills like ball control, dribbling, passing, shooting, and game tactics (Fig. 1). Throughout the intervention, the children underwent moderate-intensity training, maintaining a heart rate load of 60% to 69% of their maximum heart rate (calculated as 220 minus their age), with real-time monitoring facilitated by a Polar heart rate monitor [31]. The experimental group demonstrated an average attendance rate of 92% (range: 85–100%), with one participant exiting the study due to non-compliance. In contrast, the control group participated in non-sports activities like science, calligraphy, and painting, following the standard physical education curriculum of three 45-minute sessions per week involving general exercises such as gymnastics and relay games, without engaging in organized sports activities outside of school hours.

### Data acquisition

#### Behavioral task acquisition

**Fig. 2:**
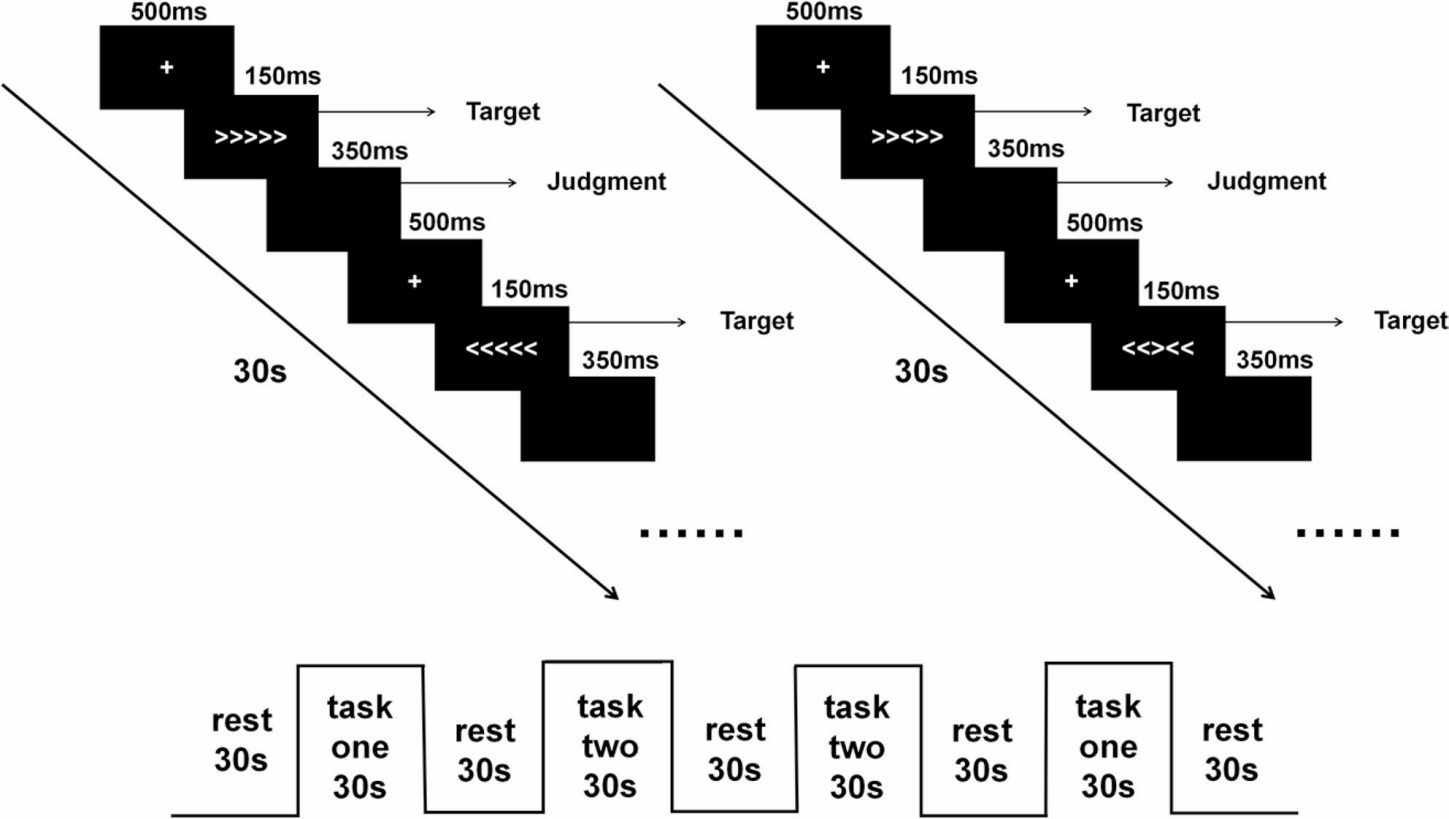
Schematic representation of the experimental design for behavioral tasks

The inhibitory control function assessed in this study was measured using the Flanker paradigm behavioral task implemented through E-prime 3.0 software. The Flanker task, originally introduced by Eriksen in 1974, is a well-established tool for measuring inhibitory control [32]. The task involved presenting stimuli in two conditions: a congruent condition (<<<<< or >>>>> ) and an incongruent condition (<<><< or >><>> ). The display resolution was set to 1366 × 768, with a black background (RGB: 0,0,0), white target color (RGB: 255,255,255), and a 24-point word size. Each trial began with a central “+” presented for 500ms, followed by the stimulus displayed for 150ms, and then a black screen shown for 350ms. Participants were instructed to respond to the direction of the target arrow (“<“ or “>”) by pressing the “F” key for “<“ and the “J” key for “>”. The task utilized a block design comprising two blocks, each lasting 30 s (Fig. 2).

#### fNIRS data acquisition

In a dimly lit classroom, participants were instructed to minimize movement of their upper body and head. The OxySoft acquisition software was utilized with the 8-channel OctaMon portable near-infrared spectral imaging system (ARTINIS, Netherlands) to observe and record blood oxygen signals in the prefrontal cortex during the execution of behavioral tasks involving the Flanker paradigm. The OctaMon device, a wearable fNIRS system, enables the continuous measurement of oxygen and deoxyhemoglobin levels in the prefrontal cortex. With eight transmitters and two receivers, the device measures near-infrared light absorption at a time resolution of 10 Hz, with the transmitters and receivers spaced 35 millimeters apart. Each emitter can emit two wavelengths (760 nm and 850 nm) simultaneously, catering to the absorption characteristics of oxygenated hemoglobin (greater absorption at wavelengths above 800 nm) and deoxygenated hemoglobin (stronger absorption at wavelengths below 800 nm).

**Fig. 3:**
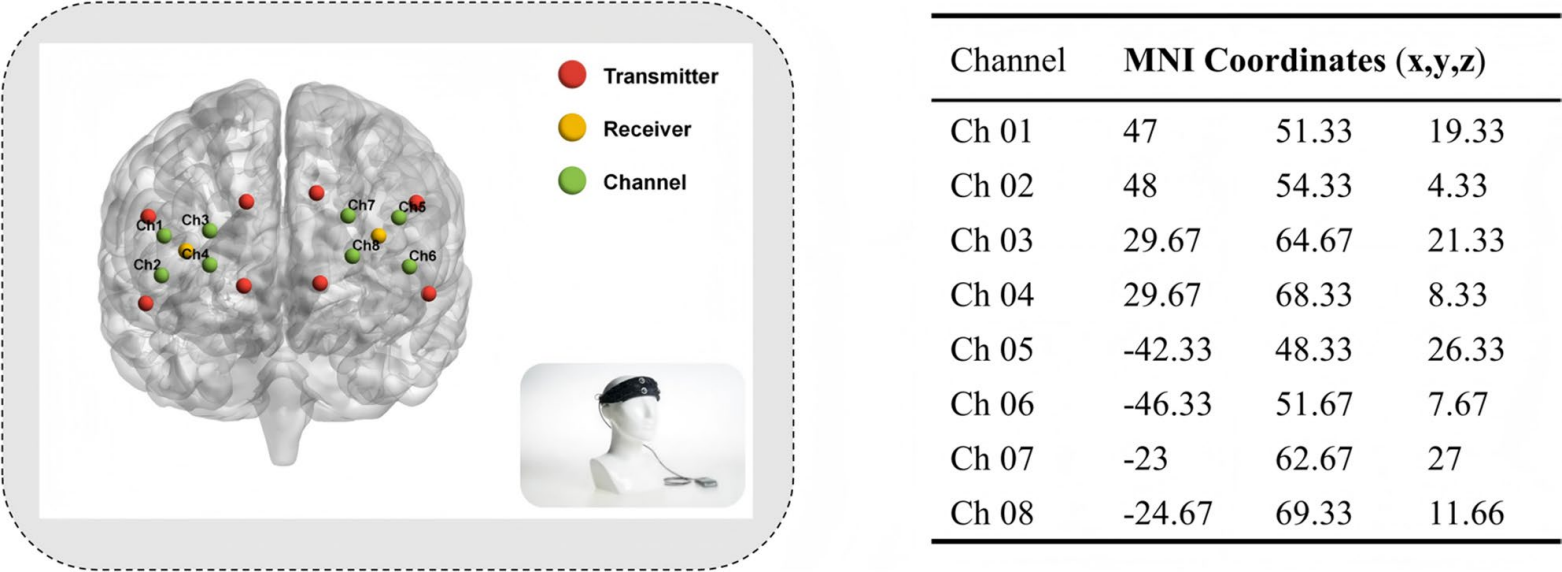
Optode arrangements for fNIRS. Eight transmitter optodes and two receiver optodes formed eight channels. Optodes were placed around the prefrontal cortexaccording to the 10–20 EEG international coordinate system

Optodes were positioned in accordance with the international 10–20 system method [33], and brain regions were delineated based on the Brodmann template. The device encompasses three Brodmann brain regions (BA10, BA11, and BA46), which are further subdivided into five regions based on regions of interest (ROI): (1) Right dorsolateral prefrontal cortex (R-DLPFC) - BA46: CH1, CH2, and CH3; (2) Left dorsolateral prefrontal cortex (L-DLPFC) - BA46: CH5, CH6, and CH7; (3) Right posterior frontal area (R-FPA) - BA10: CH3 and CH4; (4) Left posterior frontal area (L-FPA) - BA10: CH7 and CH8; (5) Orbital frontal cortex (OA) - BA11: CH4 and CH8. This configuration is illustrated in Fig. 3. Utilizing the general equation for the differential path length factor (DPF = 4.99 + 0.067*Age^0.814^) in relation to the subject’s age and the specific study protocol, changes in optical density are converted to alterations in the concentrations of O2Hb and HHb following the modified Beer-Lambert law [34, 35].

### Statistical analysis

#### Descriptive statistics for demographic data

An independent samples t-test was employed to compare the variables of age, height, weight, and body mass index (BMI) between the experimental and control groups of children, with the alpha significance level established at 0.05 as the criterion for statistical significance.

#### Statistical analysis of behavioral task data

The pre-test and post-test data of all participants were exported using E-Prime 3. Repeated measures ANOVA was conducted in SPSS 26.0 to analyze the accuracy and response time of participants in the experimental and control groups. This analysis aimed to identify differences in task performance between the groups before and after the intervention, with a significance level of α = 0.05. In cases of reciprocal benefits, a simple effect analysis was carried out to pinpoint specific differences.

#### Statistical analysis of fNIRS data

Oxygenated hemoglobin has been found to have a higher signal-to-noise ratio compared to deoxygenated hemoglobin, making it more sensitive to changes in cerebral blood flow (CBF) [36]. Some studies suggest that deoxy-Hb may reflect venous blood oxygenation and flow rather than local CBF characteristics. Therefore, this study utilized oxy-Hb as the test index [37, 38]. The fNIRS data analysis involved converting raw data using MATLAB with the oxysoft2matlab toolbox, analyzing oxygenated hemoglobin content in activated channels using the Homer2 toolbox in MATLAB. High-pass filtering at 0.008 Hz and low-pass filtering at 0.1 Hz were applied to eliminate the effects of factors such as cardiac oscillations, Mayer waves, and respiration. Optical density data was converted to concentrations of oxygenated hemoglobin, deoxygenated hemoglobin, and total hemoglobin using the modified Beer-Lambert law. Blood flow change curves were plotted to identify specific oxygenation changes. The NIRS_SPM toolbox in MATLAB was used to analyze brain response patterns during task periods based on the general linear model (GLM). Parameter estimation was conducted using the GLM model to calculate *β* values under different conditions. Repeated measures ANOVA was performed on *β* values across brain regions, with additional post-hoc analyses for any interaction effects to determine activation patterns in different brain regions.

## Research result

### Demographic characteristics

**Table 1:** Demographic characteristics of participants(M ± SD)

Variables	Experimental group (male = 20; female = 14)	Control group (male = 18; female = 17)	t	*p*
Age (years)	9.74 ± 0.86	9.54 ± 1.01	0.85	0.40
Height (cm)	149.44 ± 9.23	146.09 ± 8.07	1.61	0.11
Weight (kg)	39.29 ± 10.93	37.76 ± 4.69	0.75	0.46
BMI(kg/m^2^)	17.31 ± 3.29	17.67 ± 1.41	0.58	0.56

Prior to the experiment, demographic data were collected for all participants. t test was used to compare the demographic characteristics of the experimental group and the control group. The results showed that Age (Cohen’s d = 0.20), height (Cohen’s d = 0.39), weight (Cohen’s d = 0.18), BMI (Cohen’s d = 0.14) were not statistically significant. Table 1 lists the specific demographic information for these two groups.

### Behavioral performance

**Fig. 4:**
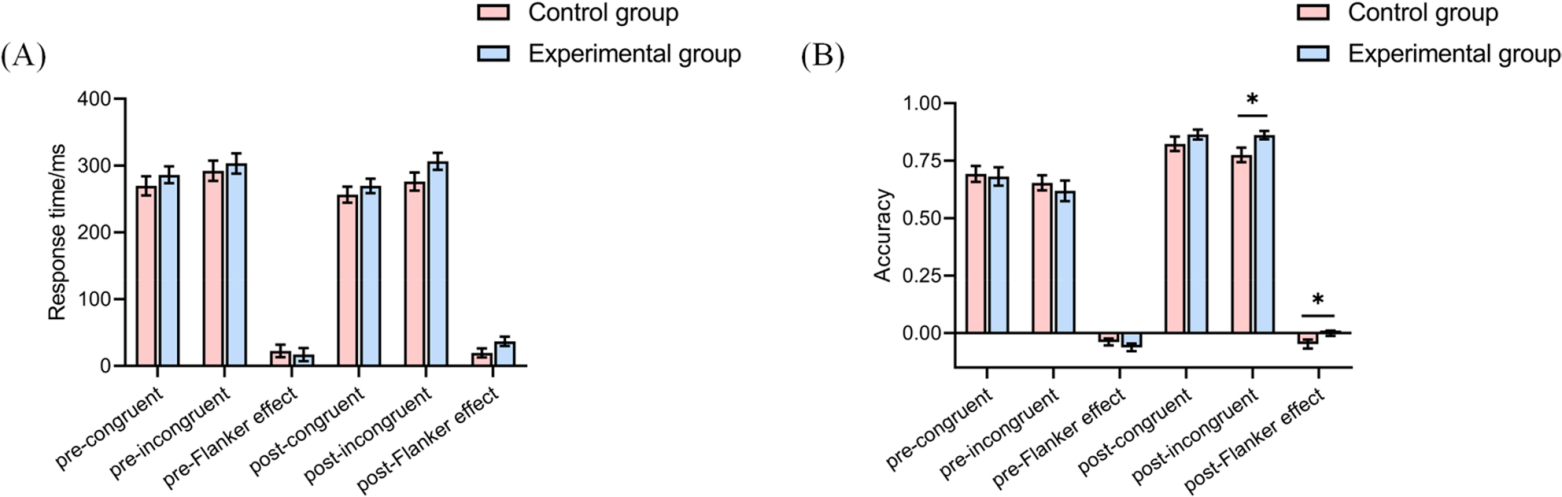
Analysis results of the behavioral tasks for the experimental group and control group. **A** Difference analysis results of response time. **B** Difference analysis results of accuracy

A repeated measures analysis was conducted on response time and accuracy within the behavioral data.The results indicate that there was no significant interaction between time-by-group in both congruent and incongruent task response times, as well as in accuracy rates for congruent tasks (Fig. 4A).This suggests that the physical exercises intervention did not significantly affect changes in task response times and simple task accuracy in children. However, for accuracy in the incongruent task, the repeated measures analysis of variance revealed a significant time × group interaction (F(1,67) = 5.487, *p* < 0.05, *η*_*p*_^2^ = 0.076], indicating a significant difference in accuracy changes in the incongruent task among school-aged children. Utilizing the Sidak correction for pairwise comparisons, a simple effects analysis revealed no significant distinction between the experimental and control groups at the pre-test stage (*p* > 0.05). However, at post-test, the experimental group displayed significantly enhanced performance relative to the control group (*p* < 0.05) (Fig. 4B).

Similarly, we conducted a repeated measures analysis of variance on the Flanker effect in terms of response time and accuracy for two groups of participants. The results showed that there was no significant time × group interaction effect on response time for the Flanker effect (Fig. 4A). However, for accuracy in the Flanker effect, there was a significant time × group interaction effect [F(1,67) = 5.340, *p* < 0.05, *η*_*p*_^2^ = 0.074], indicating a significant difference in accuracy changes in the Flanker effect for school-aged children. Following the Sidak correction for pairwise comparisons in simple effect analysis, it was observed that there was no significant distinction between the experimental and control groups before the test (*p* > 0.05). Subsequently, post-test results revealed a significant improvement in performance by the experimental group compared to the control group (*p* < 0.05). Additionally, the post-test accuracy of the Flanker effect in the experimental group was significantly higher than the pre-test (*p* < 0.05), suggesting that soccer intervention has a positive impact on children’s inhibitory control function (Fig. 4B).

### fNIRS results

**Fig. 5:**
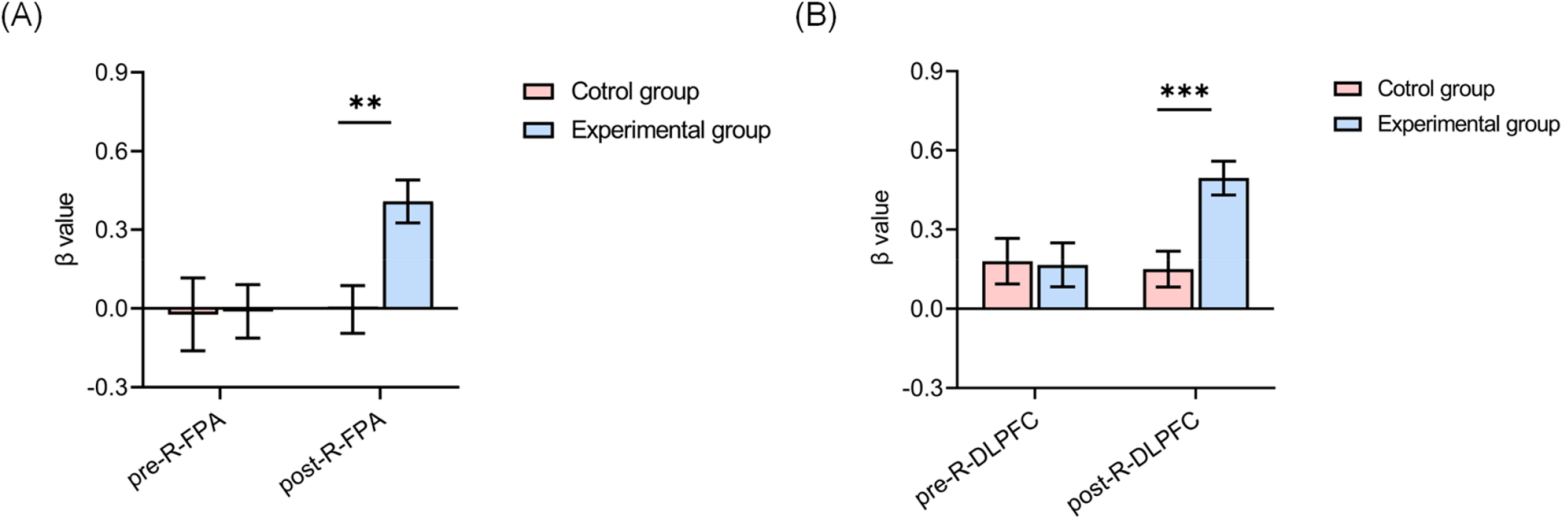
Incongruent task testing comparing *β* values in experimental group and control group. **A** Analysis of *β* values in R-FPA brain regions. **B** Analysis of *β* values in R-DLPFC brain regions

**Fig. 6:**
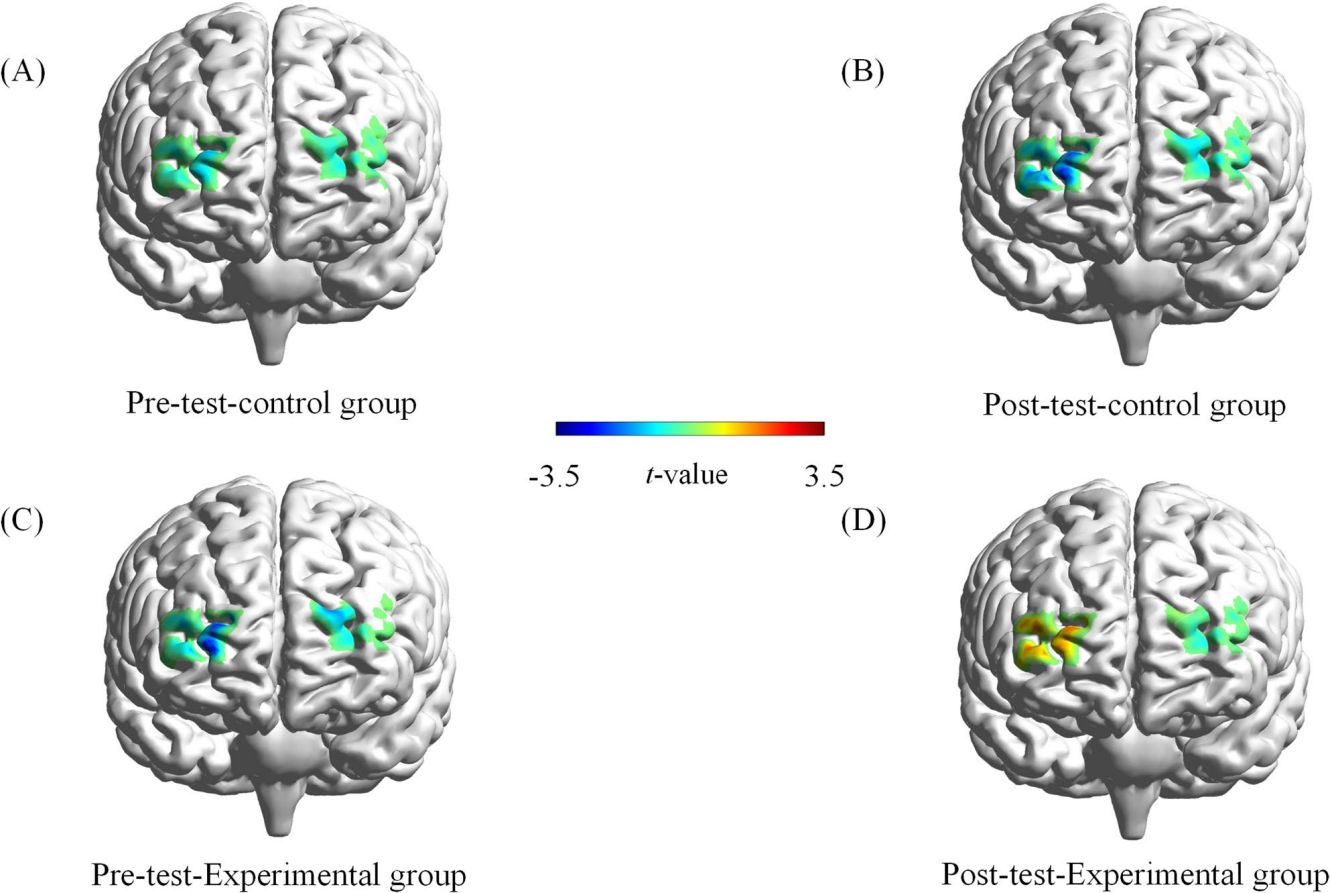
Activation maps for different channels in the prefrontal cortex. (**A**) Pre-test control group. (**B**) Post-test control group. (**C**) Pre-test experimental group. (**D**) Post-test experimental group. T-values are shown according to the color bar

To examine the variations in *β* values of cerebral oxygenated hemoglobin in distinct brain regions before and after the test, a repeated measures analysis of variance was applied. During the congruent task period, there were no significant differences in the time × group interaction effects in the R-DLPFC, L-DLPFC, R-FPA, L-FPA, and OA brain regions between the experimental and control groups (all *p* > 0.05). In the incongruent task period, there were no significant differences in the time × group interaction effects in the L-DLPFC, L-FPA, and OA brain regions between the two groups. However, significant differences were observed in the changes in the R-DLPFC [F(1,67) = 7.035, *p* < 0.05, *η*_*p*_^2^ = 0.095] and R-FPA [F(1,67) = 5.451, *p* < 0.05, *η*_*p*_^2^ = 0.075] during this period. Applying the Sidak correction for pairwise comparisons in a simple effects analysis of the cerebral hemodynamic response index *β* values in the R-DLPFC, a marked increase in post-test values compared to pre-test values was found in the experimental group (*p* < 0.01), whereas no significant alteration was noted in the control group (*p* > 0.05). Additionally, the pre-test values in the control group were higher than those in the experimental group, although this difference was not significant (*p* > 0.05). Post-test values in the experimental group were significantly higher than those in the control group (*p* < 0.001) (Figs. 5B and 6). A similar analysis on the R-FPA region showed a significant increase in post-test *β* values compared to pre-test values in the experimental group (*p* < 0.01), with no significant change in the control group (*p* > 0.05). The pre-test values were lower in the control group compared to those in the experimental group, although this difference was not significant (*p* > 0.05). The post-test values in the experimental group were significantly higher than those in the control group (*p* < 0.01) (Figs. 5A and 6).

### Correlation analysis

**Fig. 7:**
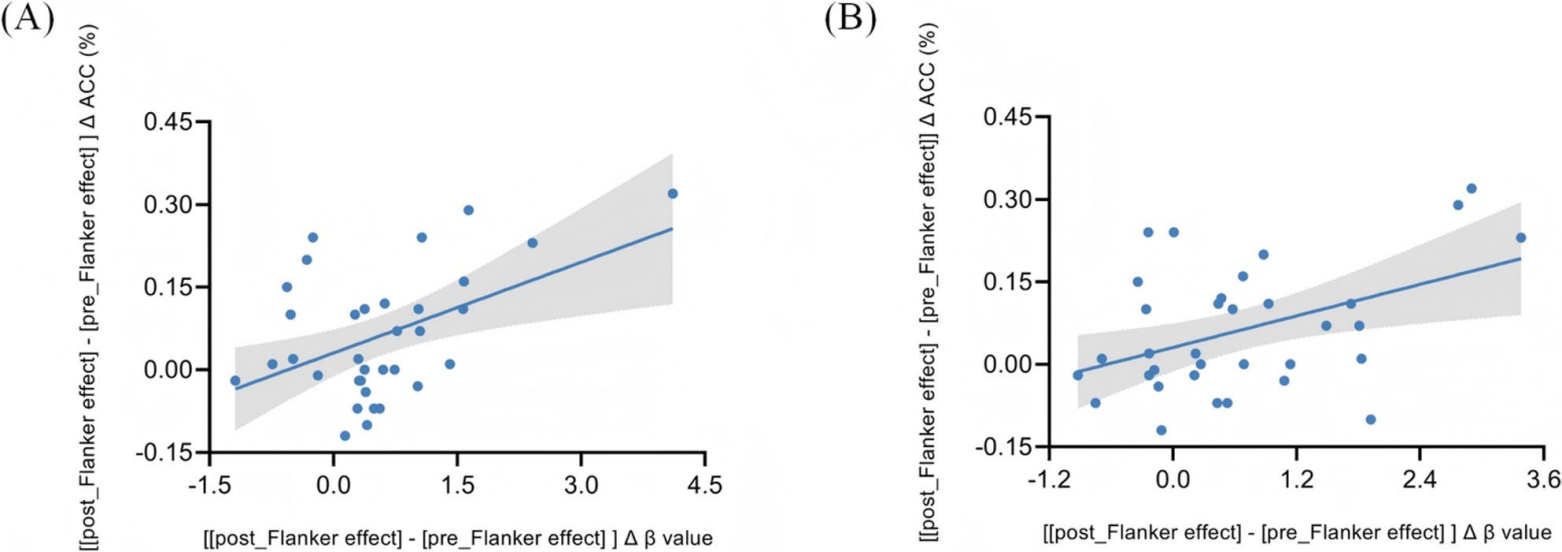
The correlation between Flanker effect and differential O2Hb signal response during flanker task in the experimental group. Graph **A** shows the correlation between O2Hb signals in ACC and R-DLPFC. Graph **B** shows the correlation between O2Hb signals in ACC and R-FPA

We conducted a two-tailed Pearson correlation analysis to investigate the association between Flanker interference accuracy and prefrontal cortex activation post-intervention. Analysis of the accuracy and O2Hb concentration difference between the incongruent-congruent post-test and pre-test tasks revealed a significant positive correlation between Flanker interference accuracy in the experimental group and O2Hb differential signals in the R-DLPFC (*r*(34) = 0.470, *p* = 0.005) and R-FPA (*r*(34) = 0.441, *p* = 0.009) regions (Fig. 7).

## Discussion

Cognitive psychology has long focused on attention and cognitive control mechanisms, particularly in children’s cognitive development. This study, conducted within the context of China’s cultural and educational milieu, investigated how continuous extracurricular football training influences children’s inhibitory control functions. The findings offer empirical support for the notion that engaging in extracurricular football activities can improve children’s inhibitory control behaviors and neurophysiological performance.

### The impact of after-school soccer exercise on children’s inhibitory control function

The intervention in this study involved a 12-week after-school soccer physical exercises program for the experimental group. The results showed that the decrease in incongruent task response time in the experimental group was significantly greater than the control group, and there was also a significant difference in the Flanker effect. This may be because the intervention strategy in this study enhanced the participants’ attention control and interference inhibitory abilities. Our findings are congruent with a study by Zhong et al., who found that after a 12-week volleyball intervention, the intervention group outperformed the control group in both accuracy and response time during the flanker task [25]. Another study demonstrated that children in the experimental group outperformed those in the control group in terms of accuracy on Stroop and flanker tests through cognitive challenging sports intervention [39]. Hence, we determine that After-school Soccer Exercise can significantly improve children’s inhibitory ability in the Chinese educational setting.

Moreover, research has demonstrated that soccer players exhibit superior inhibitory control compared to the general population [19]. This phenomenon may be attributed to the unique characteristics of the sport. Soccer is categorized as an open skill, requiring increased attention and cognitive engagement during movement execution to effectively adjust to unpredictable environmental conditions. One investigation involving 497 soccer players found a substantial positive correlation between attentional focus and inhibitory control [40]. Soccer has been proven to greatly improve children’s coordination skills. A study conducted by Alesi et al. revealed a robust positive correlation between inhibitory control and coordination through the assessment of these functions in 44 participants [41]. The intervention in this study integrates skill training with competitive elements to boost participants’ motivation and introduce greater uncertainty, thereby fostering increased cognitive engagement. This may account for the notable impact of After-school Soccer Exercise on enhancing children’s inhibitory skills. However, it is important to ascertain if this intervention’s efficacy is specific to soccer. Hence, future studies should conduct comparative assessments across different sports to elucidate the distinct role of Soccer Exercise in advancing children’s inhibitory control abilities.

Furthermore, it is important to consider that intervention and promotion strategies for children’s cognitive development are influenced by the educational system, family dynamics, and the social environment [42–44]. After a school-level physical activity intervention involving distinct groups in separate schools, both the intervention and control groups demonstrated improvements in executive control abilities within 6–8 months, as indicated by research findings. However, the enhancement in task performance among the intervention group did not exceed that of the control group [45]. Another study has demonstrated that “unconscious” aerobic exercise yields minimal improvements in EFs [46]. This may be ascribed to the societal, educational, and cultural backgrounds, along with the baseline cognitive levels of the participants [47]. The intervention framework of this study underscores the importance of viewing extracurricular football as a combined physical and cognitive activity within the Chinese school sports system. Given its relatively limited emotional and social symbolic value, this framework may elucidate the observed research outcomes.

### The impact of after-school soccer exercise on prefrontal cortex activation in children

The prefrontal lobe, particularly the right hemisphere, is recognized as a critical brain region for executive functions, encompassing attention control, decision-making, and conflict resolution [28, 48]. In this experiment, the activation level of the right prefrontal lobe in the experimental group was significantly higher than that in the control group during the task, which may be attributed to the greater mobilization of executive functions when participants engaged in the Flanker task. This finding aligns with prior research demonstrating increased prefrontal activation during tasks that require advanced cognitive processing [49]. Additionally, studies have indicated that elevated oxyhemoglobin concentrations reflect an increase in local cerebral blood flow resulting from cortical neuronal activation, suggesting enhanced cortical activation in these regions [50]. Furthermore, research has shown that aerobic exercise can effectively promote the development of children’s prefrontal lobes and executive functions [51]. In this study, through the analysis of fNIRS test results before and after the intervention, it was observed that, compared with the control group, the prefrontal lobe of the brain was significantly activated during incongruent tasks in the soccer intervention group. This phenomenon may be due to changes in local brain blood flow induced by the stimulation of relevant brain areas by behavioral tasks, which is congruent with our hypothesis.

The findings of this study demonstrated that a 12-week after-school soccer exercises intervention effectively enhanced the activation of the right dorsolateral prefrontal lobe and the right frontal polar lobe during the behavioral task (the flanker incongruent task). According to Champod et al., the right prefrontal cortex, particularly the right dorsolateral prefrontal cortex, plays a crucial role in the inhibitory control of selective attention [52]. Jiang et al. utilized fMRI to measure the brain activation levels of participants during the flanker task before and after an 8-week aerobic exercise intervention in children [53]. Their results indicated significant activation of the right prefrontal lobe during the flanker task. While cultural norms may modulate the behavioral expression of inhibitory control [44], the observed neural activation patterns likely reflect domain-general neuroplasticity mechanisms. Future cross-cultural studies should disentangle biological versus socioenvironmental effects.

Physical activity contributes to the enhancement of multiple central nervous system functions and cognitive abilities, as evidenced by a growing body of research [54]. This study suggests that engaging in open skills can activate the prefrontal cortex of children’s brains by improving certain functions and/or structures. Repeated exposure to dynamic decision-making contexts in soccer may enhance synaptic plasticity in prefrontal networks, as indicated by neuroimaging studies that associate complex motor learning with changes in gray matter [55]. Another study showed that physical activity not only enhances children’s aerobic capacity but also improves cognitive control ability by increasing the volume of the caudate nucleus [56]. Furthermore, Physical activity has been shown to stimulate individual sensorimotor coordination, increase the number of Purkinje neurons and synapses, and activate visuospatial networks associated with attention control and working memory [57]. Participation in after-school activities, particularly interactive open-ended sports, constitutes a cognitive endeavor that engages higher-order brain functions and necessitates adaptive thinking. Furthermore, physical activity has been shown to enhance brain function through the regulation of neuronal metabolism and neural plasticity, thereby improving cognitive performance in children [58, 59]. Consequently, involvement in after-school soccer can serve as an essential component of a child’s holistic development, underscoring the critical role of congruent physical activity in fostering both physical and mental growth.

### Limitations and prospects

The study employed a random allocation method, yet the assessors were not blinded to the group assignment, possibly leading to bias. Conducted in a single school environment, the study’s findings may have limited generalizability across various socioeconomic backgrounds. Unmeasured confounding factors, such as sleep patterns and parental education levels, could impact cognitive outcomes. Future research should involve multi-center trials with blinded assessments and covariate adjustments. Furthermore, the study’s use of a small number of functional near-infrared spectroscopy (fNIRS) channels focused solely on the frontal lobe, restricting the exploration of the football intervention’s effects on other brain regions in school-aged children and hindering the assessment of functional connectivity. The study also lacked specific analyses for player positions within the football intervention plan, suggesting a need for further investigation in these areas.

## Conclusion

Evidence from this study confirms that participation in a 12-week after-school soccer program leads to a significant enhancement in inhibitory control among children aged 8–11. This improvement is manifested through increased behavioral accuracy and augmented neural activity in the right prefrontal cortex, underscoring the program’s potential as a scalable cognitive intervention in China and similar cultural environments. Future investigations should delve into the long-term implications and the transferability of these findings across different cultural contexts.

## Data Availability

The data supporting this study’s findings are available from the corresponding author upon reasonable request.

## Abbreviations

fNIRS: Functional near-infrared spectroscopy
fMRI: Functional Magnetic Resonance Imaging
EEG: Electroencephalogram
R-DLPFC: Right dorsolateral prefrontal cortex
R-FPA: Right posterior frontal area
L-DLPFC: Left dorsolateral prefrontal cortex
L-FPA: Left posterior frontal area
OA: Orbital frontal cortex
GLM: general linear model
CBF: cerebral blood flow
